# Effects of L-Carnitine on the Developmental Competence of Bovine Oocytes

**DOI:** 10.3390/ani15172576

**Published:** 2025-09-02

**Authors:** Farzaneh Salek, Mohamed F. Hashem, Jacob C. Thundathil

**Affiliations:** 1 Faculty of Veterinary Medicine, University of Calgary, Calgary, AB T2N 4N1, Canada; 2 Department of Theriogenology, Faculty of Veterinary Medicine, Cairo University, Giza 12211, Egypt

**Keywords:** oocyte maturation, embryo cryopreservation, lipid content, L-carnitine, hippo signaling

## Abstract

The in vitro production of embryos has significant potential to enhance animal productivity. However, further refining of this technology is required for its widespread adoption and cost-effectiveness. The objectives were to evaluate the effects of L-carnitine (LC) on the maturation, lipid content, and Hippo signaling of oocytes, and the cryotolerance of the resulting embryos. Abattoir-derived oocytes were in vitro matured using fetal bovine serum (FBS), bovine serum albumin (BSA), or FBS + 1.5 or 3.0 mM LC. The maturation rates did not differ among FBS (83%) and FBS with LC (1.5 or 3.0 mM; 82 and 80%, respectively). In contrast, the BSA group exhibited a significantly lower maturation rate of 71% compared to the other groups. The lipid content of matured oocytes (assessed using Nile red staining) was significantly reduced in the BSA group and in the FBS + LC groups, compared to the FBS group. The blastocyst-stage embryos were cryopreserved, and their cryotolerance was evaluated by assessing their ability to re-expand and hatch after thawing. The embryos from the FBS + LC groups showed a numerically higher re-expansion rate at 24 h (78.8%), compared to the BSA (74.0%) and FBS groups (57.7%). The expression of Hippo signaling pathway genes was not significantly affected by LC, indicating that LC enhanced cryotolerance and reduced lipid content without impacting oocyte maturation or the Hippo signaling pathway.

## Simple Summary

In vitro production (IVP) of embryos is extensively used to produce progeny from elite beef and dairy cattle. However, improving the efficiency of this technology will further advance its utilization in animal production. The aims of this study were to assess the impact of L-carnitine (LC) on the oocyte lipid content, maturation, Hippo signaling, and cryotolerance of resulting embryos. Slaughterhouse-derived oocytes were in vitro matured with or without L-carnitine (LC), fertilized and cultured in vitro, and the resulting blastocyst-stage embryos were cryopreserved. Post-thaw survival of these embryos was assessed by in vitro culture. L-carnitine enhanced cryotolerance and decreased lipid content without affecting Hippo signaling or oocyte maturation.

## 1. Introduction

The expanding world population needs enhanced cattle productivity to meet rising food supply demands [1]. Current and emerging reproductive technologies are essential for improving reproductive efficiency of cattle, enabling the selection for enhanced productivity and the global distribution of superior cattle genetics [2]. In vitro production (IVP) of embryos and traditional embryo transfer (ET) are frequently employed to generate progeny from elite beef and dairy cattle. Cryopreservation allows extended preservation of embryos, which can be subsequently thawed and transferred to recipients, substantially advancing the utilization of IVP embryos [3]. Cryopreservation seeks to preserve cellular structure and function while effectively halting metabolism and enzyme activity. The two principal methods for cryopreservation are slow freezing and vitrification; both have been utilized for oocyte and embryo cryopreservation in various species [4]. The survivability of frozen-thawed IVP embryos is suboptimal, potentially attributable to modified metabolism and lipid profiles. The efficacy of cryopreservation significantly depends upon the quality of the blastocysts. Consequently, cryotolerance is greatly influenced by the lipid content in porcine and bovine embryos [5]. Cryodamage generally transpires during lipid phase transitions and lipid peroxidation processes that take place during freezing and thawing [6]. Methods to enhance the cryotolerance of IVP embryos encompass culture under hypoxic conditions to reduce oxidative stress, supplementing the culture media with antioxidants and apoptosis inhibitor [7], and strategies to expedite lipid metabolism or limit lipid buildup in the oocytes and embryos [8].

Lipids are essential biomolecules produced in the endoplasmic reticulum or acquired from the in vitro culture environment [9]. β-oxidation of lipids is crucial for fulfilling energy demands during oocyte maturation and the expansion of the blastocyst [10,11]. However, excessive or unregulated lipid metabolism can lead to oxidative stress through the generation of lipid peroxides. Lipid peroxidation may be associated with FBS supplementation, owing to the fatty acids present in FBS [12]. Embryos originating from oocytes matured in serum-supplemented media exhibited diminished quality, while the incorporation of an antioxidant (e.g., vitamin E) enhanced the rate of embryo development [13]. Optimization of the culture media is essential to diminish cytoplasmic lipid content. Lipid accumulation occurs due to the uptake of serum lipoproteins, the synthesis of triglycerides in serum, and reduced β-oxidation of lipids inside mitochondria [14]. Although lipids are essential for energy metabolism, their increased concentration in IVP embryos makes these cells more susceptible to damage caused by cryopreservation [15]. The usage of lipolytic drugs that can cleave or modify lipids, so rendering them harmless, is among the most effective and non-invasive techniques to reduce intracellular lipid content [16]. Examples of lipolytic agents encompass epinephrine, norepinephrine, isoproterenol, forskolin, and carnitine. These chemicals have been utilized to enhance lipolysis by targeting lipolysis signaling pathways [14].

This study will concentrate on carnitine among the lipolytic drugs. Carnitine is a non-proteinogenic amino acid that plays a crucial role in many energy metabolism pathways as a regulator of lipid metabolism. L-carnitine is synthesized from lysine and methionine. Carnitine is found in human follicular fluid and is linked to enhanced human fertility [17]. Since oocytes and cumulus cells are unable to biosynthesize L-carnitine from precursor amino acids [18], supplementing the culture media with L-carnitine during oocyte and embryo development may improve lipid β-oxidation and antioxidant capabilities [19]. L-carnitine functions as a cofactor for the translocation of long-chain fatty acids from the cytosol to the mitochondria [20,21]. In addition to ATP synthesis, long-chain fatty acid metabolism produces reactive oxygen species that can cause oxidative damage and diminish cryopreservation efficacy. L-carnitine is a powerful antioxidant that eliminates reactive oxygen species (ROS) [22], enhancing blastocyst development and post-thaw survival of cryopreserved in vitro produced embryos [23]. L-carnitine may enhance mitochondrial function and facilitate β-oxidation in bovine and murine oocytes, respectively [24,25]. L-carnitine improved nuclear maturation, augmented the quantity of active mitochondria in pig oocytes, diminished intracellular lipids, and facilitated preimplantation development of bovine blastocysts [10,21]. Various signaling pathways, such as Wnt, Notch, MAPK, and Hippo, play crucial roles in folliculogenesis, oocyte maturation, and embryonic development by regulating proliferation, apoptosis, and differentiation. The Hippo signaling pathway is crucial for granulosa cell proliferation, cumulus–oocyte complex development, blastocyst formation, and cell fate determination, including inner cell mass and trophectoderm differentiation [26,27,28,29].

The Hippo signaling pathway consists of mammalian sterile 20-like 1/2 (MST1/2) and large tumor suppressor kinase 1/2 (LATS1/2) as essential components [30]. The activation of MST1/2 and LATS1/2 triggers the phosphorylation of downstream effectors in the Hippo signaling pathway. Yes-associated protein (YAP) and transcriptional coactivator with PDZ binding motif (TAZ) are two essential downstream effectors of the Hippo signaling pathway, where their activation (through dephosphorylation) and inhibition (via phosphorylation) regulate the expression of downstream genes [31]. Activation of the Hippo signaling system results in the phosphorylation of YAP/TAZ, hence inhibiting their function and retaining them in the cytoplasm. In contrast, when the Hippo signaling pathway is dormant, YAP/TAZ undergo dephosphorylation and subsequently migrate to the nucleus. The activation of YAP/TAZ augments gene expression through TEAD family transcription factors. TEAD governs the transcription of crucial developmental genes and proliferation within the nucleus [32,33]. While the majority of studies regarding the significance of YAP in blastocyst formation have concentrated on murine models, new investigations have also recognized YAP as both existent and functionally pertinent in bovine blastocysts [34,35]. Despite the conservation of the Hippo signaling pathway in cattle and mice, species-specific variations may exist in the positioning of Hippo signaling components inside blastocysts [28,36].

Recent studies demonstrate that membrane phospholipids are pivotal in several intracellular signaling pathways. L-carnitine affects lipid content and composition, indicating its role in the regulation of the Hippo signaling pathway. Phospholipids, including phosphatidic acid, lysophospholipids, sphingosine-1-phosphate, and phosphoinositol, can modulate the Hippo signaling pathway by dephosphorylating and subsequently activating YAP/TAZ [33].

In conclusion, considering the previously discussed function of phospholipids in the Hippo signaling pathway and the established influence of L-carnitine on lipid metabolism regulation, the incorporation of L-carnitine into the culture media is expected to alter lipid content without compromising oocyte maturation competence via a mechanism involving the Hippo signaling pathway. This study investigated the impact of L-carnitine addition in the maturation medium on the developmental competence of oocytes. This research has the potential to enhance the outcomes of in vitro produced embryos and their cryosurvival at an interdisciplinary level.

## 2. Materials and Methods

All procedures involving animals were approved by the University of Calgary Health Sciences Animal Care Committee (“Improving the Efficiency of In Vitro Production of Embryos in Cattle,” Study #AC23-0097), in accordance with the Canadian Council on Animal Care guidelines.

### 2.1. Reagents

Unless noted, all chemicals were purchased from Sigma-Aldrich (Oakville, ON, Canada).

### 2.2. Oocyte Collection and In Vitro Maturation of Bovine Oocytes

Domestic cattle ovaries were obtained from a local abattoir (Harmony Beef Company Ltd., Calgary, AB, Canada) and transported to the laboratory in a thermos containing physiological saline at room temperature. Oocytes were aspirated from ovarian follicles into a collection tube with an 18-gauge needle attached to a vacuum pump. The collected cumulus–oocyte complexes (COCs) were examined under a microscope (Olympus, SDF, 65×, Tokyo, Japan), and only high-quality COCs with a homogenous cytoplasm and more than three layers of compact cumulus cells were selected for in vitro maturation. Across all experiments, a total of approximately 2470 bovine oocytes were collected from 550 to 600 slaughterhouse ovaries and allocated to distinct experimental groups for maturation assessment, lipid content quantification, gene expression profiling, and embryo development/cryopreservation studies. These oocytes were matured in IVM medium consisting of Hank’s medium supplemented with 0.2 mM sodium pyruvate, 0.5 μg/mL FSH, 5 μg/mL LH, 1 μg/mL estradiol, and 50 μg/mL gentamicin, as described below:

(1) FBS (control): Oocytes matured in IVM medium supplemented with 10% FBS.
(2) BSA: Oocytes matured in IVM medium without FBS and containing BSA (8 mg/mL).
(3) FBS + 1.5 mM LC: Oocytes matured in IVM medium containing 10% FBS supplemented with 1.5 mM L-carnitine.
(4) FBS + 3.0 mM LC: Oocytes matured in IVM medium containing 10% FBS and 3.0 mM L-carnitine [5,37].

IVM was performed by incubation at 39 °C in 5% CO_2_ under high humidity for 22–24 h.

### 2.3. Evaluation of Oocyte Maturation

**Figure 1:**
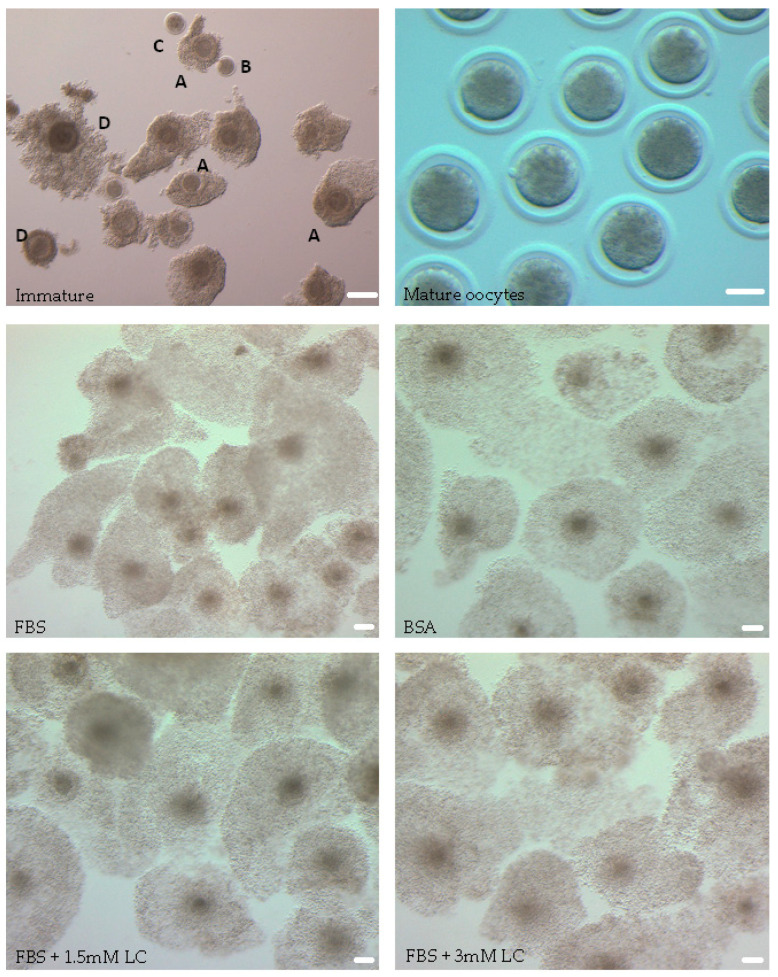
Representative images of bovine cumulus–oocyte complexes (COCs) in different treatment groups. After aspiration, COCs were examined under a light microscope and oocytes with a homogenous cytoplasm and surrounded by >3 layers of cumulus cells (A) were transferred to maturation media. Denuded oocytes (B, C), oocytes with cytoplasmic granulations (C, D), and oocytes presenting with a darkened cytoplasm (D) were considered low quality and not selected for IVM. After 24 h of IVM, COCs had substantial cumulus expansion and mature denuded oocytes with a visible polar body were observed. Scale bar = 75 µm.

IVM oocytes were cumulus dissociated (by 0.1% hyaluronidase treatment and vortexing for 1 min) and examined under a microscope (Olympus, SDF, 100×, Tokyo, Japan) for maturation status. Oocytes were classified as mature or immature, based on the presence or absence of the first polar body. A representative image of mature denuded oocytes with a visible polar body is shown in Figure 1.

### 2.4. Evaluation of the Lipid Content of In Vitro-Matured Oocytes

Nile red stock solution (1 mg/mL) was prepared by dilution in dimethyl sulfoxide (DMSO). To quantify total intracellular lipid content, IVM oocytes were washed in PBS containing 0.1% polyvinyl alcohol (PVA) and fixed in 4% paraformaldehyde. Then, these oocytes were washed 3 times in PBS-PVA 0.1% and stained with 10 μg/mL Nile red (Invitrogen, Carlsbad, CA, USA) for 4 h at room temperature. Subsequently, oocytes were washed 3 times in PBS-PVA 0.1%, mounted on glass slides, and examined by confocal microscopy (Leica, SP8, 63×, Buffalo Grove, IL, USA). ImageJ Software (version 1.54f) was used to quantify mean fluorescence intensity of oocytes to assess their lipid contents [24,38].

### 2.5. In Vitro Fertilization

To confirm maturation status of oocytes after treatment, IVF and embryo culture were performed. Frozen semen straws were thawed in a 37 °C water bath and semen quality was verified. A swim-up technique was used to prepare and capacitate sperm. The swim-up media contained sodium hyaluronate (Vetoquinol, Fort Worth, TX, USA) and fertilization media (1:1), which were incubated at 39 °C and 5% CO_2_. Thawed semen (250 μL) was loaded in a sealed Pasteur pipette placed inside a microtube and gently overlaid with 100 μL of pre-warmed and equilibrated swim-up media, followed by 200 μL of fertilization media, and then placed in an incubator for 45 min at 39 °C and 5% CO_2_. After swim-up, 150 μL of upper fertilization media was aspirated, assessed for sperm motility, and used for IVF. In vitro-matured oocytes from the above experimental groups were assessed under a microscope and those with expanded cumulus cells and a homogenous cytoplasm were transferred to pre-equilibrated fertilization drops. Oocytes were equilibrated in fertilization drops for ~1 h, then incubated with sperm as prepared above (~1–2 × 10^6^ sperm/mL of fertilization medium) from a bull with proven fertility.

### 2.6. In Vitro Embryo Culture

Following 18 h of sperm–oocyte co-incubation, all presumptive zygotes (fertilized oocytes) were cumulus dissociated by vortexing for 1 min in HEPES-buffered synthetic oviduct fluid (H-SOF) medium (107.7 mM NaCl, 7.16 mM KCl, 25.07 mM NaHCO_3_, 1.19 mM KH_2_PO_4_, 1.5 mM sodium lactate, 1.5 mM Glucose, 10 mM HEPES, 3 mg/mL BSA, 10 μM EDTA, 0.33 mM sodium pyruvate, 1 mM L-Glutamine, 1X MEM non-essential amino acid, and 50 μg/mL gentamicin) containing 0.1% hyaluronidase. Cumulus-dissociated presumptive zygotes were washed in H-SOF and synthetic oviduct fluid (SOF) media. Zygotes from each group were allocated and cultured in SOF media [107.7 mM NaCl, 7.16 mM KCl, 25.07 mM NaHCO_3_, 1.19 mM KH_2_PO_4_, 1.5 mM sodium lactate, 1.5 mM glucose, 1.71 CaCl_2_⋅2H_2_O, 0.49 mM MgCl_2_⋅6H_2_O, 8 mg/mL BSA, 0.33 mM sodium pyruvate, 1 mM L-Glutamine, 1X MEM non-essential amino acids, 1X MEM essential amino acids, and 50 μg/mL gentamicin with 2.5% FBS and TCH serum replacement (Protide Pharmaceuticals, Crystal Lake, IL, USA)]. All groups were incubated at 39 °C in a humidified atmosphere of a gas mixture of 5% O_2_, 5% CO_2_, and 90% N_2_ until day 7. Embryos were evaluated on day 2 for cleavage (un-cleaved versus cleaved) and day 7 for blastocyst production (blastocyst versus no blastocyst), based on International Embryo Transfer Society criteria [39].

### 2.7. Cryopreservation of Embryos by Slow Freezing

For cryopreservation, day 7 blastocysts were washed in holding media (BoviPro, MOFA Global, Verona, WI, USA) and transferred to the freezing medium (BoviPro, MOFA Global) [40]. Each blastocyst was loaded into a 0.25 mL straw, after 5 min of total exposure of embryos to freezing medium. These straws were placed in a programmable freezer (Beltron EFT 3002, Longmont, CO, USA) maintained at −6.5 °C. After holding for 1 min, these straws were seeded by touching the holding medium at both the top and bottom ends of straws with a cotton swab dipped in liquid nitrogen. Then straws were maintained in the chamber of the programmable freezer at −6.5 °C for >10 min, then cooled to −35 °C at a controlled rate (0.5 °C/min), plunged, and stored in liquid nitrogen. To evaluate cryotolerance, embryos were thawed for 1 min in a water bath (37 °C). Blastocysts were washed, cultured in SOF media. and at 24 and 48 h assessed under a microscope for re-expansion (yes/no) and hatching (yes/no).

### 2.8. Gene Expression Analysis

To evaluate gene expression, oocytes matured in media containing FBS with or without L-carnitine were collected at the end of each treatment. Pools of ~70 oocytes were first cumulus-dissociated, then washed 3 times in PBS-PVA 0.1% and subsequently transferred to extraction buffer (50 μL) at 42 °C for 10 min, snap frozen in liquid nitrogen, and stored at −80 °C. An Arcturus PicoPure RNA isolation kit (Applied Biosystems by Thermo Fisher Scientific, Vilnius, Lithuania) was used to extract RNA according to manufacturer’s instructions. After RNA extraction, an iScript reverse transcription kit was used for cDNA synthesis, according to manufacturer’s instructions. Subsequently, real-time quantitative polymerase chain reaction was performed on cDNA to assess expression of target genes, with expression normalized to *PPIA* (*Peptidylprolyl isomerase A*) as a reference gene [41]. The Bio-Rad CFX96 real-time PCR was used to perform qPCR. To confirm maturation status of oocytes, the expression of selected maturation markers *Cyclin-dependent kinase 1* (*CDK1*) and *Cyclin B1* genes were quantified from matured oocytes using qPCR. Also, the expression of Hippo signaling pathway components (*MST1*, *MST2*, *LATS1*, *YAP*, and *Tead-4*) was assessed by qPCR. To test primer efficiency, RNA was extracted from bovine testis tissue. A standard curve was generated for each primer using a 10-fold serial dilution of testis-derived cDNA. Threshold cycle (CT) values were plotted against the log10 of the cDNA input, and efficiency (E) was calculated based on the slope of the standard curve using the following formula:

$$E=10^{\left(-\frac{1}{slope}\right)}-1$$

The RT-qPCR target genes and their primer efficiencies in the bovine testis are in Table 1.

### 2.9. Statistical Analyses

Statistical analyses were performed using GraphPad Prism 10.2.2 and GraphPad Prism software version 2024.9.0.375. Results are mean ± SEM unless otherwise indicated. All experiments were replicated 3–6 times to generate the required numbers of matured oocytes. For categorical data with a single replicate or pooled replicates (e.g., re-expansion and hatching of embryos), a Chi-square test of independence was performed. When the Chi-square test indicated a statistically significant group effect, post hoc pairwise comparisons were conducted using Fisher’s exact test with Bonferroni correction. For categorical data with multiple replicates (e.g., polar body formation, cleavage, blastocyst formation), a binomial regression model was used to account for replicate-to-replicate variability and differing sample sizes per replicate. When the binomial regression indicated a statistically significant group effect, post hoc pairwise comparisons were conducted using Tukey’s HSD on replicate-level proportions. For continuous data (e.g., mean fluorescence intensity), normality within each group was assessed using Shapiro–Wilk tests, and homogeneity of variances was evaluated using Levene’s test. If assumptions for parametric analysis were met, linear mixed-effects models with treatment group as a fixed effect and replicate as a random effect were used to account for within-replicate correlation and unequal sample sizes. When the estimated random effect variance was negligible, data were summarized to replicate-level means and analyzed using one-way ANOVA followed by Tukey’s post hoc test for pairwise comparisons. For gene expression data, the log_2_ fold-change values were assumed to be non-normal because one group had zero variance (all normalized values equal to 1). A two-tailed Mann–Whitney U test was used to compare log_2_ fold-change values between groups, as it does not require normality and remains valid when one group has no variability.

## 3. Results

### 3.1. In Vitro Oocyte Maturation Status

#### 3.1.1. Cumulus Expansion and First Polar Body Formation

The maturation status of oocytes from all experimental groups was evaluated based on first polar body formation and morphological assessment of cumulus cells. Expanded cumulus cells can be considered a morphological indicator of oocyte maturation, as they assist meiotic resumption of the oocyte [42,43]. After 24 h of in vitro maturation, cumulus–oocyte complexes belonging to all experimental groups had cumulus expansion, with no visible morphological differences among groups (Figure 1) when examined under a microscope (Olympus, SDF, 40×). The mean percentages of mature oocytes with polar body formation in both the 1.5 and 3.0 mM L-carnitine treatment groups (82.0 and 80.0%, respectively) were comparable to the FBS-only group (83.2%) (Table 2). However, the BSA group had a significantly lower maturation rate (70.6%) compared to the FBS group and the groups supplemented with L-carnitine (*p* < 0.01).

Differences in the proportion of oocytes exhibiting first polar body extrusion among four treatment groups were analyzed using a binomial regression model. For each replicate (*n* = 6 per group), the numbers of First PB and No PB oocytes were used directly. The analysis showed a significant overall effect of treatment group on the probability of First PB extrusion (*p* < 0.001). Post hoc pairwise comparisons (Tukey’s HSD on replicate-level proportions) indicated that BSA had significantly lower First PB proportions than FBS (*p* = 0.0027), FBS + LC 1.5 mM (*p* = 0.0126), and FBS + LC 3 mM (*p* = 0.0285). No significant differences were found among FBS, FBS + LC 1.5 mM, and LC 3 mM (all *p* > 0.71). Values with different superscripts (a, b) differ (*p* < 0.05). *N* = 6 replicates. *n* = 34–65 oocytes per group per replicate, with a total of 286–312 oocytes per group when replicates are combined.

#### 3.1.2. Expression of Oocyte Maturation Markers

**Figure 2:**
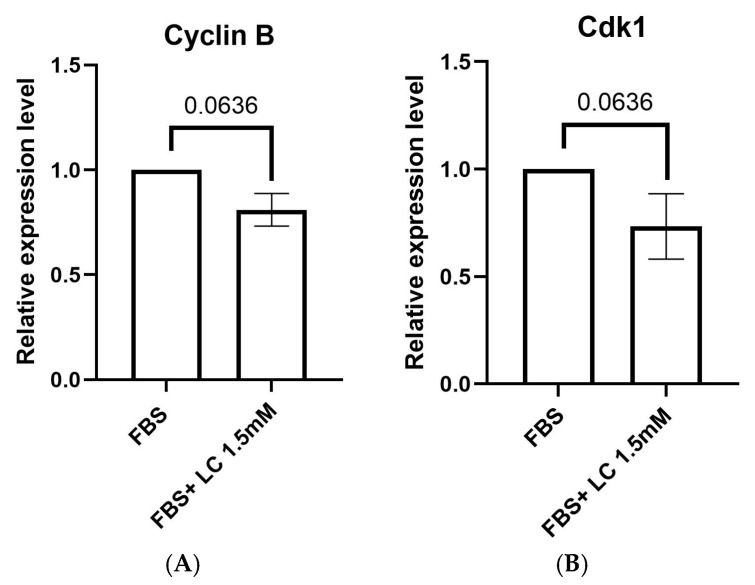
Mean ± SEM expression of maturation marker genes in oocytes matured in media containing FBS and FBS + L-carnitine (1.5 mM). Expression levels of (**A**) *Cyclin B* (*p* = 0.0636) and (**B**) *Cdk1* (*p* = 0.0636) in bovine oocytes measured by quantitative real-time PCR. A Mann–Whitney U test was used for statistical analysis. FBS: Fetal bovine serum, LC: L-carnitine. ns = not significant, *N* = 3 replicates. *n* = 60–70 pooled oocytes per replicate per group, with a total of 180–210 pooled samples per group when replicates are combined.

*Cyclin B* and *cyclin-dependent kinase 1* (*Cdk1*) are candidate genes involved in resumption of meiosis and the maturation of oocytes. Expression levels of *Cyclin B* and *Cdk1* genes were similar (*p* > 0.05) between oocytes matured in media supplemented with or without 1.5 mM L-carnitine (Figure 2).

### 3.2. Lipid Content of Matured Oocytes

**Figure 3:**
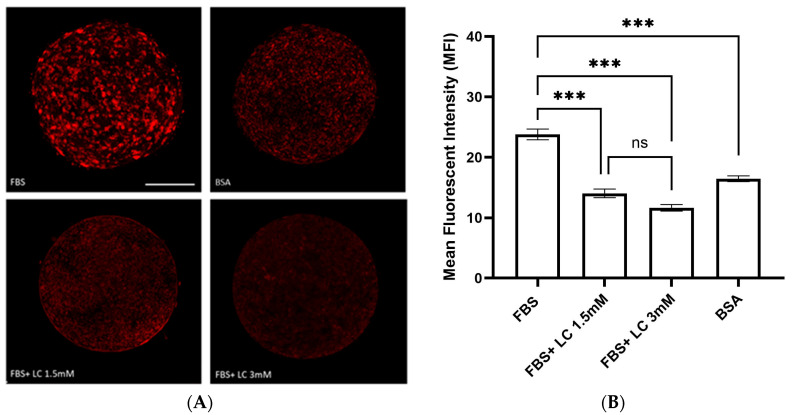
Relative amounts of lipid in bovine oocytes. (**A**) Representative images of cytoplasmic lipid droplets stained with Nile red in matured oocytes. (**B**) Quantification of lipid content expressed by mean fluorescence intensity (MFI) per area (mean ± SEM). The data were initially analyzed using a linear mixed-effects model (LMM) with treatment group as a fixed effect and replicate as a random effect. Assumption checks performed on the raw data indicated no evidence of non-normality within groups (all Shapiro–Wilk *p* > 0.13) but did reveal heterogeneity of variances between groups (Levene’s test, *p* = 0.0126). To account for this, a heteroscedastic model was fitted using weighted least squares (WLS) with group-specific residual variances. Using FBS as reference, the WLS model estimated that BSA (−7.32, *p* < 0.001), LC 1.5 (−9.76, *p* < 0.001), and LC 3 (−12.14, *p* < 0.001) all had significantly lower MFI values. As the random effect variance from the LMM was effectively zero, data were then summarized to replicate-level means (*n* = 4 per group) and analyzed using one-way ANOVA, which confirmed a significant group effect (F (3, 12) = 34.37, *p* = 0.000004). Tukey’s HSD post hoc test on replicate means showed that FBS was significantly higher than all other groups (all *p* < 0.001), and LC 1.5 and LC 3 did not differ significantly (*p* = 0.058). Assumption checks for the ANOVA indicated no violations: residuals were visually consistent with a normal distribution (QQ plot) and displayed constant spread across fitted values (residuals vs. fitted plot), with statistical tests supporting these observations (Shapiro–Wilk *p* = 0.566, Levene’s test *p* = 0.752). FBS: fetal bovine serum, BSA: bovine serum albumin, LC: L-carnitine. Scale bar = 75 μm. *** *p* < 0.001, ns = not significant. *N* = 4 replicates. *n* = 6–13 oocytes per group per replicate, with a total of 39–43 oocytes per group when replicates are combined.

Oocytes matured in media containing FBS had the highest mean fluorescent intensity (23.7 MFI; Figure 3), indicating significant (*p* < 0.001) accumulation of lipid droplets compared to all other groups. In contrast, the mean fluorescent intensity of oocytes matured in BSA-containing media was 16.4, a significant reduction (*p* < 0.001) in lipid content relative to the FBS group. Adding L-carnitine (1.5 or 3.0 mM) to FBS-containing maturation medium reduced lipid content to 14.0 and 11.6 MFI, respectively, compared to the FBS-only group (*p* < 0.001). No significant differences in fluorescence intensity were observed between the 1.5 mM and 3.0 mM L-carnitine groups (*p* > 0.05).

### 3.3. In Vitro Fertilization and Embryo Production

The competence of oocytes to develop into embryos following maturation in different types of media was evaluated. Table 3 indicates the progression from cleaved embryos to fully developed blastocysts by day 7. The cleavage rates of the FBS and FBS + 1.5 mM L-carnitine groups were comparable (67.3 and 69.3%, respectively). The cleavage rate of the BSA group (57.3%) was lower than the FBS and FBS + 1.5 mM L-carnitine groups (*p* < 0.05), but the differences were not statistically significant. Rates of blastocysts derived from oocytes matured with FBS or FBS + 1.5 mM L-carnitine were similar (31.8 and 31.6%, respectively). However, the rate of blastocyst development in the BSA group was significantly reduced (22.4%) compared to the other groups (*p* < 0.05).

Differences in the proportion of oocytes that cleaved and the proportion of cleaved embryos forming blastocysts among three treatment groups (FBS, BSA, and LC 1.5 mM) were analyzed using separate binomial regression models. For each replicate, the numbers of cleaved/uncleaved oocytes and blastocyst/non-blastocyst embryos were used directly. The analysis for cleavage did not show a significant overall effect of treatment group on the probability of cleavage, and post hoc pairwise comparisons (Tukey’s HSD on replicate-level proportions) indicated no significant differences among groups (all *p* > 0.05). Nevertheless, the comparison between BSA and LC 1.5 mM yielded a *p* value of 0.0624, indicating a trend toward lower cleavage proportions in the BSA group. In contrast, the analysis for blastocyst formation showed a significant overall effect of treatment group (*p* < 0.001), with post hoc comparisons indicating that BSA had significantly lower blastocyst proportions than FBS (*p* = 0.0008) and LC 1.5 mM (*p* = 0.0010), whereas FBS and LC 1.5 mM did not differ significantly (*p* = 0.9931). Values with different superscripts (a, b) differ (*p* < 0.05). *N* = 6 replicates, *n* = 42–52 oocytes per group per replicate, with a total of 217–232 oocytes per group when replicates are combined.

### 3.4. Post-Thaw Survival of Cryopreserved Embryos

**Figure 4:**
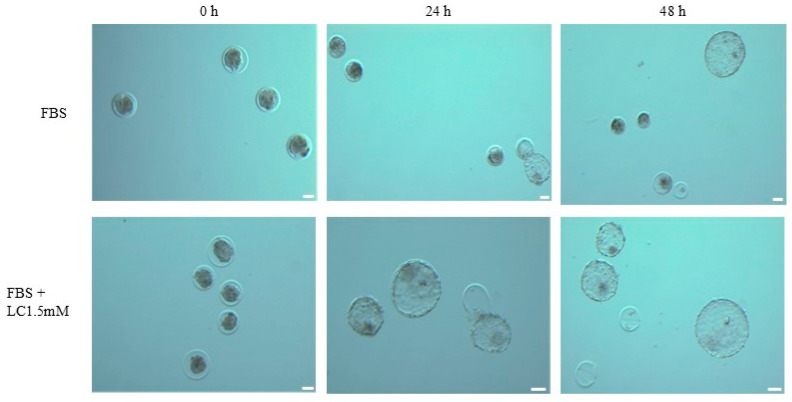
Representative images showing the progression of frozen-thawed embryos derived from oocytes matured in media containing FBS or FBS supplemented with 1.5 mM L-carnitine (LC) at 0 h, 24 h, and 48 h post-thaw. At 0 h, embryos immediately after thawing display a contracted structure as a result of the cryopreservation process. By 24 h and 48 h post-thaw, embryos in both groups have fully re-expanded and, in some cases, hatched. Scale bars = 75 μm.

Embryos from oocytes matured in media containing BSA, FBS, or FBS with L-carnitine were cryopreserved. Re-expansion and hatching rates of these embryos after thawing are shown in Figure 4 and Table 4. At 24 h post-thaw, embryos derived from oocytes matured in medium supplemented with FBS + 1.5 mM L-carnitine had the highest re-expansion rate (78.8%), showing a non-significant trend toward an increase compared to the FBS group (57.7%). The BSA group had a re-expansion rate of 74.0%, not significantly different from the other two groups. At 48 h post-thaw, there were no significant differences in re-expansion rate among all the groups, although the FBS + L-carnitine group showed a trend toward a higher re-expansion rate than those in the FBS (82.7 and 63.5%, respectively) or BSA (76.0%) groups. Hatching rates between groups at both 24 and 48 h post-thaw were similar.

Re-expansion and hatching outcomes were evaluated at both 24 h and 48 h following thawing. At 24 h, differences in the proportion of embryos that re-expanded among the three treatment groups (BSA, FBS, and LC 1.5 mM) were analyzed using a Chi-square test of independence, which showed a statistically significant overall effect of treatment group on the probability of re-expansion (χ^2^ = 6.083, df = 2, *p* = 0.0478). Post hoc pairwise comparisons were performed using Fisher’s exact test, with *p*-values adjusted using the Bonferroni correction (adjusted α = 0.05/3 = 0.0167). None of the pairwise comparisons reached statistical significance after correction (BSA vs. FBS: raw *p* = 0.0979, adjusted *p* = 0.2937; BSA vs. LC 1.5 mM: raw *p* = 0.6436, adjusted *p* = 1; FBS vs. LC 1.5 mM: raw *p* = 0.0343, adjusted *p* = 0.1030), although the comparison between FBS and LC 1.5 mM showed a non-significant trend toward a higher re-expansion rate in the LC 1.5 mM group. Hatching rates at 24 h, calculated among embryos that had re-expanded, were also compared among the three groups and showed no significant overall effect (χ^2^ = 0.165, df = 2, *p* = 0.9209). All post hoc pairwise comparisons were far from statistical significance (adjusted *p*-values = 1). At 48 h, re-expansion analysis did not show a statistically significant overall effect of treatment group (χ^2^ = 5.151, df = 2, *p* = 0.0761). Post hoc Fisher’s exact tests similarly showed no significant differences after correction (BSA vs. FBS: raw *p* = 0.1999, adjusted *p* = 0.5997; BSA vs. FBS + LC 1.5 mM: raw *p* = 0.4675, adjusted *p* = 1; FBS vs. FBS + LC 1.5 mM: raw *p* = 0.0455, adjusted *p* = 0.1366), although a non-significant trend toward a higher re-expansion rate in the FBS + LC 1.5 mM group compared to FBS was observed. Hatching rates at 48 h, calculated among embryos that had re-expanded, showed no significant overall effect (χ^2^ = 0.250, df = 2, *p* = 0.8823), with all post hoc comparisons far from significance (adjusted *p*-values = 1). Embryos originated from six independent replicate sources, and at the end of each replicate embryos were cryopreserved and subsequently thawed together for analysis. There was a total of 50–52 embryos per group.

### 3.5. Expression of Hippo Signaling Pathway Component Genes

**Figure 5:**
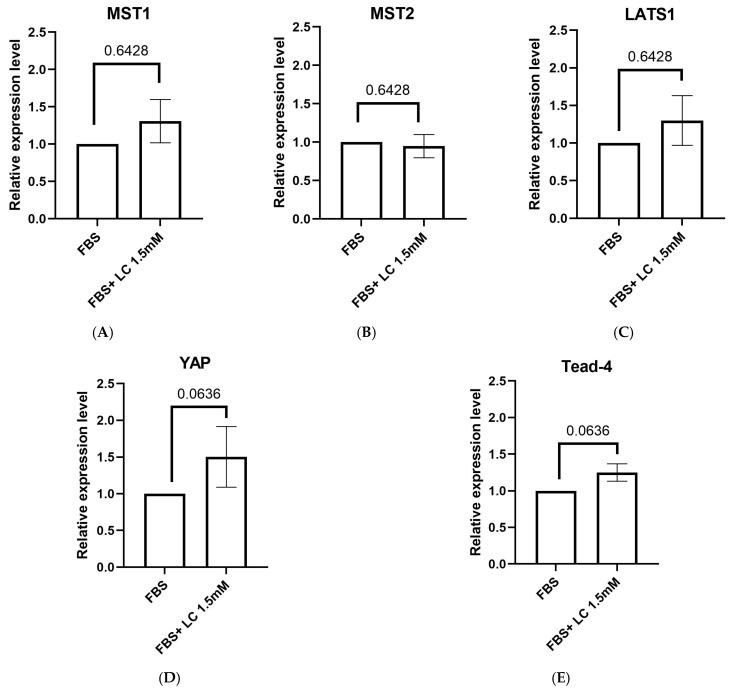
Mean ± SEM expression of components of the Hippo signaling pathway genes in oocytes matured in media containing FBS, with or without 1.5 mM L-carnitine. Expression levels of (**A**) *MST1* (*p* = 0.6428), (**B**) *MST2* (*p* = 0.6428), (**C**) *LATS1* (*p* = 0.6428), (**D**) *YAP* (*p* = 0.0636), and (**E**) *Tead-4* (*p* = 0.0636) in bovine oocytes measured by qPCR. Replicates = 3. Data was analyzed using a Mann–Whitney U test. *p* > 0.05, ns = not significant. *n* = 60–70 pooled oocytes per replicate per group, with a total of 180–210 pooled oocytes per group when replicates are combined.

In order to compare the expression levels of different Hippo pathway components, quantitative PCR (qPCR) was performed on oocytes matured in media containing only FBS or FBS + 1.5 mM L-carnitine. The FBS-only group served as the control and was used to normalize the gene expression levels measured in the L-carnitine group. The expression levels of *MST1* (1.3 ± 0.2), *MST2* (0.9 ± 0.1), and *LATS1* (1.2 ± 0.3) were not different between the FBS-only and FBS + L-carnitine groups in matured bovine oocytes (*p* > 0.05). Additionally, the expression levels of the downstream effector of the Hippo signaling pathway *YAP* (1.5 ± 0.4) and its transcription factor *Tead-4* (1.2 ± 0.1) were not different between the two groups (Figure 5). Nevertheless, there was a trend of increased expression of *YAP* and *Tead-4* (*p* = 0.0636) in the FBS + L-carnitine group compared to the FBS-only group.

## 4. Discussion

The objectives of this study were to: (1) compare effects of maturation media components (FBS, FBS + L-carnitine, and BSA) on maturation status and lipid content of bovine oocytes, their developmental competence and cryotolerance of resulting embryos; (2) investigate effects of L-carnitine supplementation in FBS-containing maturation media on the Hippo signaling pathway in bovine oocytes.

The higher maturation rate of oocytes in the FBS group may be related to the wide range of components in FBS (e.g., vitamins, growth factors, cytokines, hormones, antioxidants) [44,45]. These components can promote resumption of meiosis and oocyte development. Beneficial effects of FBS on oocyte maturation and embryo development have been reported in several animal species [46,47]. Despite its widespread use, some studies have raised concerns regarding the use of FBS as a standard media supplement due to its variability; therefore, ongoing research is focused on identifying a reliable alternative [48]. BSA is one of the most common supplements used as an alternative source of FBS for culture systems. It has been demonstrated that both FBS and BSA enhanced oocyte maturation and stimulated protein synthesis within oocytes, although oocytes matured in FBS-supplemented media had higher rates of protein synthesis, which is essential for meiotic resumption [49]. Effective use of growth factors such as EGF and FGF2 as alternatives for FBS and BSA in culture systems has been reported [50]. In the present study, oocyte maturation rates were comparable between media containing FBS alone and FBS with L-carnitine, with no significant differences between the two tested L-carnitine concentrations (1.5 and 3.0 mM). Additionally, expression of *Cyclin B* and *Cdk1* in oocytes matured in FBS and FBS supplemented with L-carnitine indicated successful maturation, as these genes have essential roles in cell cycle regulation and progression through meiosis, confirming that both media supported oocyte maturation. Consistent with these results, L-carnitine treatment improved nuclear maturation and elevated *Cdk1* and *Cyclin B* gene expression in matured mouse oocytes, with no significant differences between 1.5 mM and 3 mM L-carnitine [51].

Serum supplementation in the maturation media has been associated with alterations in oocyte metabolism and the accumulation of lipid in the oocytes, which can reduce embryo viability, particularly cryo-survival [52]. To improve IVP efficiency and embryo quality, numerous components, including L-carnitine, have been tested to optimize media for oocyte maturation and embryo culture. Adding L-carnitine in the maturation medium significantly reduced cytoplasmic lipid content [53]. Similarly, we observed that oocytes matured with FBS had a high lipid content in the cytoplasm based on Nile red staining. However, oocytes matured in the IVM medium supplemented with FBS + L-carnitine or BSA had significantly reduced intracytoplasmic lipid content compared to those matured in IVM medium with FBS alone. Although our findings indicated that elimination of FBS and supplementation of maturation media with BSA could be beneficial, as BSA resulted in lower lipid content than FBS, rates of maturation and embryo development were significantly lower than other groups, attributed to the elimination of growth factors and other beneficial effects of FBS. Carrillo-González et al. [52] reported that supplementation of 3.8 mM L-carnitine during oocyte maturation altered lipid metabolism and significantly decreased lipid content. In contrast, in another study there were not changes in the lipid content of oocytes matured with L-carnitine, although these authors reported alterations in the localization of lipids in these oocytes [24]. Causes of variations among findings are unclear, although differences in species, media, incubation time, as well as temperature and gas concentration may have contributed.

To verify developmental competence, oocytes matured under various conditions were fertilized and cultured in vitro, and their subsequent embryo development was evaluated. Consistent with our maturation status results, there were no significant differences in cleavage and blastocyst rates in oocytes matured in IVM medium supplemented with FBS alone or in combination with L-carnitine (1.5 mM) compared to BSA. Similarly, Shirazi et al. reported there were higher cleavage and blastocyst formation rates in sheep oocytes matured with FBS compared to BSA. However, blastocysts derived from FBS had reduced cryotolerance following cryopreservation, and they suggested that FBS during in vitro maturation of oocytes may have adversely affected the cryosurvival of resulting embryos due to cytoplasmic lipid content [54]. In vitro-produced embryos typically have elevated lipid content and altered lipid profiles, likely due to suboptimal culture conditions and metabolic imbalances, which can compromise developmental competence and cryotolerance. Alterations in post-thaw survival ability can reduce pregnancy rates after embryo transfer, compared to embryos developed in vivo. Studies suggested that oocytes and embryos cultured in serum-containing media are apparently more sensitive to chilling and freezing due to increased lipid content. However, reducing lipid content by using serum-free media decreased lipid content and enhanced cryotolerance [55,56]. Consistent with these data, in our study, oocytes matured in FBS had a lower rate of re-expansion at 24 h and the addition of L-carnitine was associated with a numerically higher re-expansion rate, suggesting a possible improvement in cryotolerance. In contrast, another study reported that L-carnitine supplementation during oocyte maturation did not have a significant impact on embryo development or re-expansion rates of resulting embryos; however, when L-carnitine was added during embryo culture, re-expansion rates were increased [37]. Another study explored effects of varying concentrations of FCS (2.5, 5, 7.5, and 10%) in combination with L-carnitine (1.5 mM) on embryo development and survival after cryopreservation. Their results revealed that embryo culture media with L-carnitine and lower FCS concentrations (2.5 or 5%) had a higher production rate and improved cryosurvival compared to higher FCS concentrations [57]. Supplementation of L-carnitine during oocyte maturation showed a trend toward higher re-expansion rates of cryopreserved embryos at 24 h compared to those matured with FBS alone, suggesting a potential reduction in intracellular lipid content and a possible improvement in cryotolerance. However, the trend of increased hatching rates was not significant, possibly because hatching involves additional factors beyond lipid content, e.g., enzymatic activity and structural changes in the zona pellucida, which L-carnitine may not influence [34].

In a study comparing the effects of in vitro and in vivo embryo production on lipid composition, IVP embryos had significant alterations in their lipid profile, especially lysophosphatidylcholine (LPC) concentrations [14]. This study suggested that reduced quality and survival of IVP embryos may be caused through these lipid profile changes, which could subsequently impact the Hippo signaling pathway, a crucial regulator of cellular development and viability [14]. Another study demonstrated that phosphatidic acid could regulate the Hippo signaling pathway through direct lipid–protein interactions and suggested that lipids and their alterations could impact cellular behavior, providing potential therapeutic insights by targeting the Hippo signaling pathway [58]. Additionally, L-carnitine has a key role in facilitating fatty acid oxidation, stabilizing phospholipids and influencing lipid homeostasis [17,59]. Lipid metabolism can regulate cellular signaling pathways including the Hippo signaling pathway, which is crucial for ovarian follicular development, oocyte maturation, and embryo development [26,60]. Thus, we aimed to explore the potential regulatory effects of L-carnitine, through its involvement in lipid metabolism, on the expression of the Hippo signaling pathway components in matured oocytes.

Most studies on the Hippo signaling pathway focus on protein expression and post-translational modifications, with only a few investigating mRNA expression levels. However, assessing transcriptional regulation could offer valuable insights into how upstream signals and environmental cues shape pathway activity. Thus, we compared the mRNA expression levels of core components *MST1*, *MST2*, and *LATS1*, as well as downstream effectors *YAP* and *TEAD-4*, in oocytes matured in FBS alone versus FBS with L-carnitine. Gene expression analyses confirmed the presence of Hippo signaling components in matured oocytes. There was a numerical (albeit not significant) increase in *MST1* and *LATS1* expression in oocytes matured in the FBS + L-carnitine group compared to the FBS group; however, the expression level of *MST2* was similar between groups. In addition, there was a non-significant upward trend in the mRNA expression of *YAP* and its transcription factor *Tead-4* in the L-carnitine group. Overall, there were no statistically significant variations in the expression levels of Hippo signaling pathway components between oocytes matured in FBS and those supplemented with L-carnitine. The lack of differences among groups may be due to numerous factors provided by FBS in maturation media that can regulate the Hippo signaling pathway through various pathways during oocyte maturation [33,61,62]. Thus, supplementation of L-carnitine in maturation media may have a limited influence on the transcriptional regulation under these conditions, indicating that FBS alone provides a complex regulatory environment. This aligns with previous findings that FBS components can regulate different signaling pathways, including the Hippo signaling pathway. For instance, growth factors such as insulin-like growth factor 1 (IGF1) and ligands from the epidermal growth factor (EGF) family can regulate the Hippo signaling pathway by activating receptor tyrosine kinases in cancer cells, which can stimulate other signaling pathways like phosphoinositide 3-kinase (PI3K) and AKT and lead to YAP activation [61,63]. Moreover, studies have shown that serum acting through G protein-coupled receptors resulted in YAP’s nuclear localization via a mechanism mediated by PI3K and PDK1 [33].

Regulation of the Hippo signaling pathway is highly complex and varies among cell types and conditions. This pathway can be regulated by a multitude of signals and factors such as mechanical forces, extracellular matrix interactions, cell–cell adhesion, cell polarity, G protein-coupled receptors, and cellular metabolic processes [30,33,64]. Additionally, studies have shown that, aside from the Hippo kinase cascade, several non-Hippo kinases are involved in regulating YAP and influencing cellular functions. Kinases from other pathways, e.g., mTOR, MAPK, NF-κB, also have a role in YAP regulation. These kinases can modulate YAP/TAZ activity in response to various stimuli or under specific conditions [65,66,67,68]. Therefore, L-carnitine supplementation may not directly affect these upstream regulatory signals. Instead, it may act on downstream components, perhaps indirectly through other protein kinases and signaling pathways such as metabolic pathways, leading to a nonsignificant change in Hippo signaling pathway gene expression in matured oocytes. Under energy stress, AMP-activated protein kinase (AMPK) directly phosphorylates and inactivates YAP, reducing its nuclear localization and transcriptional activity. Conversely, with energy sufficiency such as enhanced lipid metabolism and ATP production AMPK activity decreases, potentially allowing YAP to remain active and support its transcriptional processes. Accordingly, L-carnitine supplementation supports the hypothesis of enhanced lipid metabolism and its contribution to ATP production, which may, in turn, reduce AMPK activation. By lowering AMPK activity, L-carnitine may regulate its downstream transcriptional activity and prevent YAP phosphorylation [69].

A study demonstrated that phospholipid lysophosphatidic acid (LPA) accelerates blastocyst formation by influencing the Hippo signaling pathway. In bovine embryos cultured with LPA, gene expression analysis revealed no significant differences in *YAP* transcription compared to controls in day 6 and day 8 blastocysts. *TAZ* and *TEAD4* gene expressions were significantly upregulated in day 6 blastocysts treated with LPA, but no differences observed in day 8 blastocysts. Despite the lack of change in *YAP* gene expression, YAP level was elevated in day 6 and day 8 embryos exposed to LPA. These findings suggest that LPA does not affect *YAP* transcription but instead enhances YAP stability by inhibiting its degradation [70]. In the current study, there was no significant effect of L-carnitine on Hippo signaling gene expression. Further research, including advanced lipidomics, mass spectrometry, and analysis of protein-level changes or post-translational modifications, is needed to explore its potential role in modulating lipid profiles and Hippo signaling during oocyte maturation.

## 5. Conclusions

In conclusion, maturation media influenced oocyte maturation and their developmental competence. Adding FBS in IVM medium supported oocyte maturation and promoted developmental competence. However, FBS resulted in excessive lipid accumulation in oocytes, reducing the cryotolerance of resulting embryos. Using BSA in the IVM medium instead of FBS reduced the intracytoplasmic lipid content of oocytes and improved the cryotolerance of resulting embryos, but compromised oocyte’s ability to mature and develop to blastocysts. In oocytes matured in media containing FBS with L-carnitine there was less intercellular lipid, due to L-carnitine enhancing lipid metabolism. Furthermore, these oocytes had a similar rate of maturation and subsequent embryonic development compared to those matured in media containing FBS, with enhanced embryo cryotolerance. In addition, L-carnitine did not alter the expression level of the hippo signaling pathway. These findings underscored the importance of optimizing in vitro maturation conditions to enhance developmental competence and highlighted the need for further exploration into the regulation of the Hippo pathway and other potential pathways that could be modulated by this treatment.

Future investigations could explore whether L-carnitine indirectly modulates the Hippo signaling pathway by targeting other signaling factors. Considering L-carnitine’s role in lipid metabolism, future studies could examine its influence on other lipid-associated signaling pathways (e.g., AMPK, mTOR) and their potential crosstalk with the Hippo signaling pathway. Whereas this study focused on gene expression, future studies would evaluate how L-carnitine affects the Hippo signaling pathway at the protein level or through post-translational modifications, which could reveal their regulatory mechanisms.

## Figures and Tables

**Table 1:** Primer sequences, and efficiencies of bovine targets genes. Primer efficiency was evaluated using a standard curve method on bovine testis tissue.

Target	Accession Number	Primer Sequence	Primer Efficiency (%)
*PPIA*	NM_178320.2	TCTTGTCCATGGCAAATGCTG	99.29
		TTTCACCTTGCCAAAGTACCAC	
*MST1*	NM_001075677.2	CCCACTTGCCTGCTTTACTC	104.07
		GTCGGACAGAAAGCTTCAGG	
*MST2*	NM_001079607.2	AACGGATACAATGGCGAAAC	102.87
		GTTGGTGGTGGGTTTGTAGG	
*LATS1*	NM_001192866.1	CAGCAGCTGCCAGACCTATTA	103.83
		TCCAGCTCTGTTTGCGGTTA	
*YAP*	XM_015466436.1	ACGGTGCTTTGACCTAATCG	104.48
		ATGCCACCCAATACAACCAG	
*TEAD-4*	XM_059887105.1	TCCACAGTAAAATGACCCTCACC	101.131
		GTGTAGGTTTGCTGGGAGAAAG	
*Cyclin B*	NM_001045872.1	CATGGAAACATCTGGCTGTG	98.96
		TGACTGCTTGCTCTTCCTCA	
*CDK1*	L26547.1	TGTGCTTATGCAGGATTCCA	99.37
		GGCCAAAATCTGCCAACTTA	

**Table 2:** Evaluation of oocyte maturation after 24 h of IVM by first polar body quantification.

Group	Total *n*	Oocytes with Polar Body *n* (%)
FBS	305	254 (83.2) ^a^
FBS + LC 1.5 mM	312	256 (82.0) ^a^
FBS + LC 3 mM	310	248 (80.0) ^a^
BSA	286	202 (70.6) ^b^

**Table 3:** Cleavage and blastocyst rates of oocytes matured in media containing FBS, FBS + L-carnitine (1.5 mM), or BSA.

Group	Total *n*	Cleaved Embryos *n* (%)	Blastocysts *n* (%)
FBS	217	146 (67.3) ^a^	69 (31.8) ^a^
FBS + LC 1.5 mM	225	156 (69.3) ^a^	71 (31.6) ^a^
BSA	232	133 (57.3) ^a^	52 (22.4) ^b^

**Table 4:** Re-expansion and hatching rates of embryos derived from oocytes matured in media containing BSA, FBS, or FBS supplemented with L-carnitine after cryopreservation at 24 and 48 h post-thaw.

24 h				48 h	
Group	Total	Re-Expansion *n* (%)	Hatching *n* (%)	Re-Expansion *n* (%)	Hatching *n* (%)
FBS	52	30 (57.7)	10 (33.3)	33 (63.5)	12 (36.4)
FBS + LC 1.5 mM	52	41 (78.8)	15 (36.6)	43 (82.7)	17 (39.5)
BSA	50	37 (74.0)	12 (32.4)	38 (76.0)	13 (34.2)

## Data Availability

Data are contained within the article.
